# Effect of Rapid Thermal Annealing on the Characteristics of Micro Zn-Doped Ga_2_O_3_ Films by Using Mixed Atomic Layer Deposition

**DOI:** 10.3390/nano15070499

**Published:** 2025-03-26

**Authors:** Jiajia Tao, Xishun Jiang, Aijie Fan, Xianyu Hu, Ping Wang, Zuoru Dong, Yingjie Wu

**Affiliations:** 1 Shanghai Microwave Technology Research Institute (No. 50 Research Institute of China Electronics Technology Group Corporation), Shanghai 200331, China; 2 Zhangjiang Laboratory, Shanghai 201210, China; 3 State Key Laboratory of ASIC and System, School of Microelectronics, Fudan University, Shanghai 200433, China; 4 School of Mechanical and Electronical Engineering, Chuzhou University, Chuzhou 239000, China

**Keywords:** micro Zn-doped Ga_2_O_3_ film, atomic layer deposition, thermal annealing effect, electrical property

## Abstract

In this work, micro Zn-doped Ga_2_O_3_ films (GZO) were deposited by one-step mixed atomic layer deposition (ALD) followed by post-thermal engineering. The effects of Zn doping and post-annealing temperature on both structure characteristics and electric properties were investigated in detail. The combination of plasma-enhanced ALD of Ga_2_O_3_ and thermal ALD of ZnO can realize the fast growth rate (0.62 nm/supercyc.), high density (4.9 g/cm^3^), and smooth interface (average R_q_ = 0.51 nm) of Zn-doped Ga_2_O_3_ film. In addition, the thermal engineering of the GZO was achieved by setting the annealing temperature to 400, 600, 800, and 1000 °C, respectively. The GZO film annealed at 800 °C exhibits a typical crystalline structure (Ga_2_O_3_: β phase, ZnO: hexagonal wurtzite), a lower roughness (average R_q_ = 2.7 nm), and a higher average breakdown field (16.47 MV/cm). Notably, compared with the pure GZO film, the breakdown field annealed at 800 °C increases by 180%. The O_V_ content in the GZO after annealing at 800 °C is as low as 34.8%, resulting in a remarkable enhancement of electrical properties. These research findings offer a new perspective on the high-quality ALD-doped materials and application of GZO in high-power electronics and high-sensitive devices.

## 1. Introduction

In recent years, the next-generation wide bandgap semiconductor material gallium oxide (Ga_2_O_3_) has shown great potential applications in power electronic devices and solar-blind ultraviolet photo-detectors due to its superior characteristics, including a larger bandgap (4.6–4.9 eV), a higher breakdown field strength (~8 MV/cm), and superior chemical and thermal stability [1,2,3]. At present, researchers have improved the optical, electrical, photoelectric, or optoelectrical properties of Ga_2_O_3_ materials through doping, noble metal modification, or energy band engineering. Among the doping, modification, and energy band engineering, doping engineering is the simplest and most effective method to enhance the performance of Ga_2_O_3_-based devices [4]. However, the related precise control of electrical properties for Ga_2_O_3_ thin films by doping engineering is still a huge challenge. It was found that Si-doped β-Ga_2_O_3_ thin films developed through MOCVD can only non-uniform line dope controlled by employing pulse width modulation [5]. In addition, the correlation between structure, chemistry, and dielectric properties of iron-doped Ga_2_O_3_ were investigated roughly by the standard high-temperature solid-state chemical reaction method [6]. Moreover, the Zr-doping strategy for Cu_2_O/Ga_2_O_3_ (Ga_2_O_3_/ZrO_2_ = 98.7:1.3 wt%) was investigated to achieve a high-performance solar-blind ultraviolet photodetector through magnetron sputtering deposition [7]. In summary, conventional synthesis methods face significant challenges in achieving large-area fabrication, highly uniform deposition, and precise trace doping of Ga_2_O_3_ thin films due to inherent limitations in traditional equipment capabilities and intricate reaction kinetics, which are essential for effectively tailoring their electrical and optoelectronic properties.

Atomic layer deposition (ALD) has emerged as a suitable technique for achieving precise control over both thickness and compositional uniformity in surface engineering, particularly in conformal coating applications and in situ doping processes of nanostructured semiconductors. While numerous studies have demonstrated the successful deposition of Ga_2_O_3_ thin films using ALD methodology, research focusing on controlled elemental doping strategies in Ga_2_O_3_ via ALD remains comparatively limited. Moreover, the window temperature of the ALD growth of Ga_2_O_3_ is below 400 °C, in which Ga_2_O_3_ films are in an amorphous or polycrystalline state [8]. These amorphous or polycrystal states contain a large number of uncertain grain boundaries, defects, and oxygen vacancies, which greatly hinder the electric or photoelectric performance of the device [9]. Accordingly, the thermal engineering after the ALD processing of Ga_2_O_3_ film is an important issue for improving photoelectric performance [10]. In addition, Zn atoms have similar atomic radii to Ga atoms, so they can be used for the most efficient doping control [11]. Tao et al. investigated the morphological, electrical, and optical properties of the Zn-doped Ga_2_O_3_ amorphous film, including the change of film density and resistivity, roughness, transmittance, and bandgap [12]. Singh et al. achieved green light emission and p-type characteristics of ZnGaO films [13]. Hu et al. prepared Zn: Ga_2_O_3_ films by co-injecting sALD with predictable doping content and realized a high-performance photodetector [14]. However, the post-thermal engineering of Zn element micro doped-Ga_2_O_3_ during the ALD process is still lacking systematic research. Moreover, the basic mechanisms of the doping and post-thermal effect are still absent, which hinders the basic understanding of the ALD doping effect and the exploration of new performances of Ga_2_O_3_-based devices.

Herein, the Zn-doped Ga_2_O_3_ thin films were grown by mixed ALD, in which the Zn: Ga were precisely adjusted by a fixed cycle of thermal processing of ZnO and a plasma-enhanced process of Ga_2_O_3_. The subsequent post-thermal engineering of thin films grown by ALD was achieved by rapid annealing at 400 °C, 600 °C, 800 °C, and 1000 °C for 5 min in an N_2_ atmosphere. The ALD micro-doping of the Zn element and thermal engineering effects on optical, structural, and electrical properties of Ga_2_O_3_ film were disused in detail. The annealed GZO film shows superior crystallinity, high transparency, low surface roughness, and a high breakdown field. This micro dopant and post-thermal treatment process successfully improved the optical and electrical properties of GZO film, which broadens its potential applications in photodetection and high-power devices.

## 2. Materials and Methods

Zn-doped Ga_2_O_3_ films were deposited on Si substrates by using the ALD system (BENEQ TFS200, Espoo, Finland). Trimethylgallium (TMG) and diethylzinc (DEZn) were used as Ga and Zn precursors. Oxygen plasma and H_2_O were used as the precursors for Ga_2_O_3_ and ZnO. All the precursors in the experiment were weighed using a METTLER TOLEDO ME 204 balance. The deposited temperatures during all ALD processes were kept at 200 °C. The specific parameters of ALD-deposited films are listed in the Supplementary Information. After ALD processing, the samples were annealed at 400, 600, 800, and 1000 °C, respectively, in an N_2_ atmosphere for 5 min through rapid thermal processing (Annealsys, Montpellier, France, AS-One). The thickness of the deposited GZO films was measured by spectroscopic ellipsometry (SE, SOPRA GES-5E). X-ray diffraction (XRD, D8 Advance, Bruker, Billerica, MA, USA) was used to examine the crystallinity of the films. Meanwhile, the film density was fitted using X-ray reflectivity (XRR, D8-Advance, Bruker). The surface roughness of GZO films was checked with atomic force microscopy (AFM, Bruker). X-ray photoelectron spectroscopy (XPS, Thermo Scientific, Waltham, MA, USA) was conducted to analyze the chemical composition and state. The MOS device fabrication details are shown in the Supplementary Information. The current-voltage (I–V) characteristics of the GZO-based MOS devices were measured by a Keithley 4200 semiconductor parameters test system.

## 3. Results and Discussion

**Figure 1:**
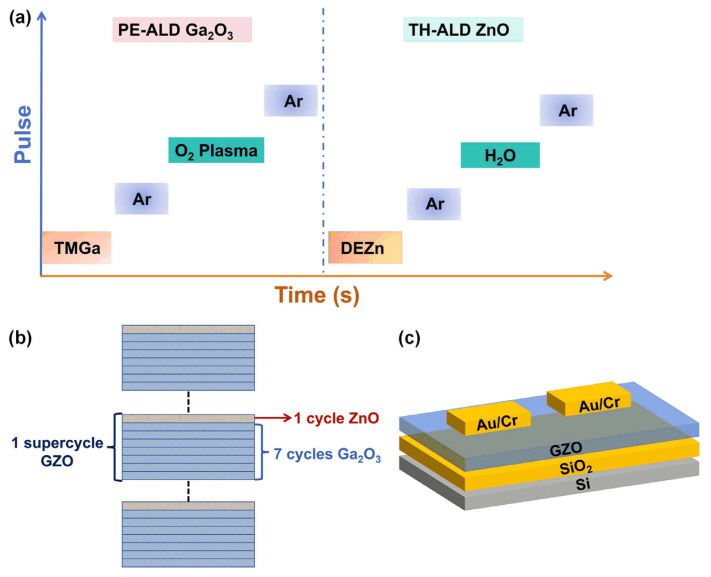
(**a**) GZO thin film deposition process, including Ga_2_O_3_ and ZnO monolayer deposition process; (**b**) schematic diagram of GZO laminated film structure consisting of seven layers of Ga_2_O_3_ and one layer of ZnO, and (**c**) MOS device structure based on GZO structure.

The cycle ratio of Ga_2_O_3_ and ZnO with the same film thickness was precisely regulated and compared by using the unique atomic layer film deposition characteristics of ALD, and a super cycle of Zn-doped Ga_2_O_3_ films containing seven cycles of Ga_2_O_3_ and one cycle of ZnO was designed. For better differentiation, the sample prepared after several super cycles is named GZO. A supercycle process and the structure diagram of GZO deposited by ALD are shown in Figure 1a,b. Accordingly, the structure of the GZO based on the MOS device after photolithography, metal deposition, and stripping is shown in Figure 1c.

**Figure 2:**
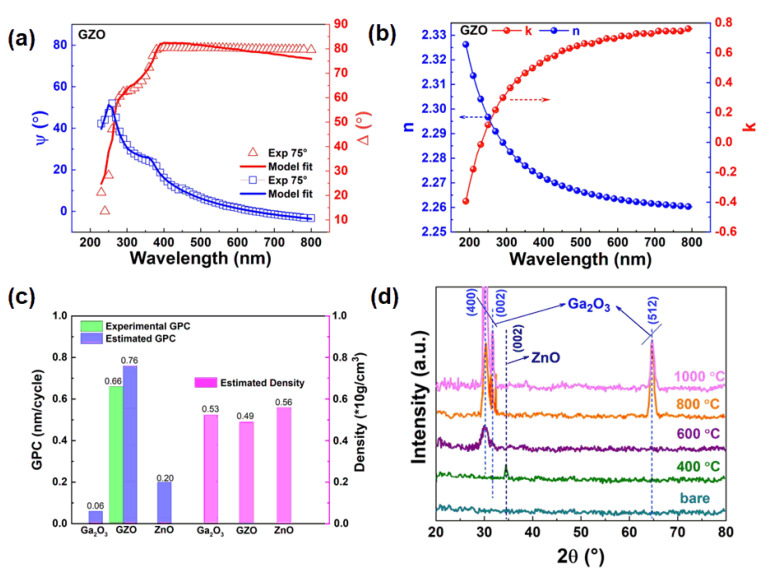
(**a**) SE test and fitting curves of polarization angles and phase angles, (**b**) the change of refractive index n and extinction coefficient k with wavelength of original GZO films, (**c**) a supercyclic theoretical superposition and measured deposition rate of GZO, Ga_2_O_3_, and ZnO films, (**d**) XRD patterns of GZO films with different rapid annealing temperatures.

In order to obtain the thickness, refractive index (n), extinction coefficient (k), and density of the films, SE was used to systematically measure the micro Zn-doped Ga_2_O_3_ films deposited by ALD. The corresponding results are shown in Figure 2a–c. Figure 2a shows the SE test and fitting curves of the polarization angle and phase angle of the GZO thin film. Experimental results are highly consistent with calculated results in the test wavelength range, indicating the high accuracy of fitting results. The thickness of the GZO film is 20 nm through the calculation and fitting of the Cauchy model. As shown in Figure 2b, in the wavelength range of 230–800 nm, with the increase in wavelength, the refractive index of GZO film became smaller and maintained normal dispersion characteristics. At the wavelength of 632.8 nm, the n value of the film is 2.26, which is higher than that of GZO films prepared by traditional methods [15,16], indicating that GZO films prepared by ALD have the advantages of dense and high electron density. At the same time, the extinction coefficient of all films decreases with the increase in wavelength and is closer to zero at 500–800 nm, indicating that GZO has a high transmittance in the visible light region [17]. This suggests that ALD shows great advantages in depositing transparent electrode films due to their high density and good transmittance. Figure 2c shows the growth rate for Ga_2_O_3_, ZnO, and GZO films. By comparison, it is found that the deposition rate of GZO film (0.76 nm/cyc.) is much higher than the superposition deposition rate of the ZnO and Ga_2_O_3_ thin films (0.62 nm/supercyc.). The design of GZO is based on a supercycle consisting of seven PE-ALD deposition Ga_2_O_3_ cycles and one TH-ALD deposition ZnO cycle. The estimated and measured thickness of ZGO film are listed in Table S1. It shows that the scheme realizes the rapid growth of Zn-doped Ga_2_O_3_ thin films. In addition, Figure 2c also shows the different film density values obtained after SE fitting. The densities of Ga_2_O_3_, GZO, and ZnO films are 5.3, 4.9, and 5.6 g/cm^3^, respectively. The GZO thin film shows an amorphous state. It can be concluded that the decrease of thin film density is mainly attributed to the corresponding defects in the internal Ga_2_O_3_ thin films caused by Zn doping [18].

Figure 2d shows the XRD pattern of GZO films on Si substrate before and after the annealing process. In the bare GZO films, no other diffraction peaks are found, which indicates that the Zn-doped Ga_2_O_3_ film is amorphous. The peaks of the Si substrate are shown in Figure S1 for better comparison. However, according to a previous study [12], the ZnO films deposited by TH-ALD have a hexagonal wurtzite crystal structure, unlike the amorphous state of deposited Ga_2_O_3_ film by PE-ALD. Obviously, the amorphous state of Ga_2_O_3_ film leads to the poor crystallinity of the GZO film. In particular, the change of crystallinity of Zn-doped Ga_2_O_3_ films deposited in this mixed ALD mode is different from that of other ALD-doped composite films. For example, during the ALD process of Al-doped ZnO, the crystallinity of AlZnO films depends on the Al content [19]. The crystalline properties of doped films depend on the preparation method, intrinsic property, and post-thermal engineering. Accordingly, the (400) and (002) peaks of β-Ga_2_O_3_ in GZO film exhibit the highest diffraction intensity after a 600 °C annealing process [20]. As the annealing temperature increases, the peak intensity of Ga_2_O_3_ rises, and the FWHM becomes narrower. This indicates that high temperature promotes the crystallization and growth of the film. The reason is that the high-temperature annealing treatment at the appropriate temperature enables the gallium atoms and oxygen atoms inside the film to obtain sufficient energy to migrate to the appropriate positions and eliminate the oxygen vacancies inside the film. In addition, a typical crystalline hexagonal wurtzite structure of ZnO with (002) peak is shown in the GZO before and after annealing. At low annealing temperatures (≤ 400 °C), Zn may act as a nucleation site, lowering the energy barrier for crystallization. This can lead to an earlier onset of crystallization compared to undoped Ga₂O₃. In addition, Zn might stabilize metastable Ga₂O₃ polymorphs at lower temperatures due to lattice strain or defect interactions, delaying the transition to the stable β-phase. At high annealing temperatures ( ≥ 600 °C), Zn doping could accelerate β-Ga₂O₃ formation by facilitating atomic rearrangement despite initial strain from the larger Zn^2+^ ions. Also, exceeding Zn solubility limits may result in ZnO precipitates, which could either pin grain boundaries or act as templates for epitaxial growth, depending on interfacial compatibility [21].

**Figure 3:**
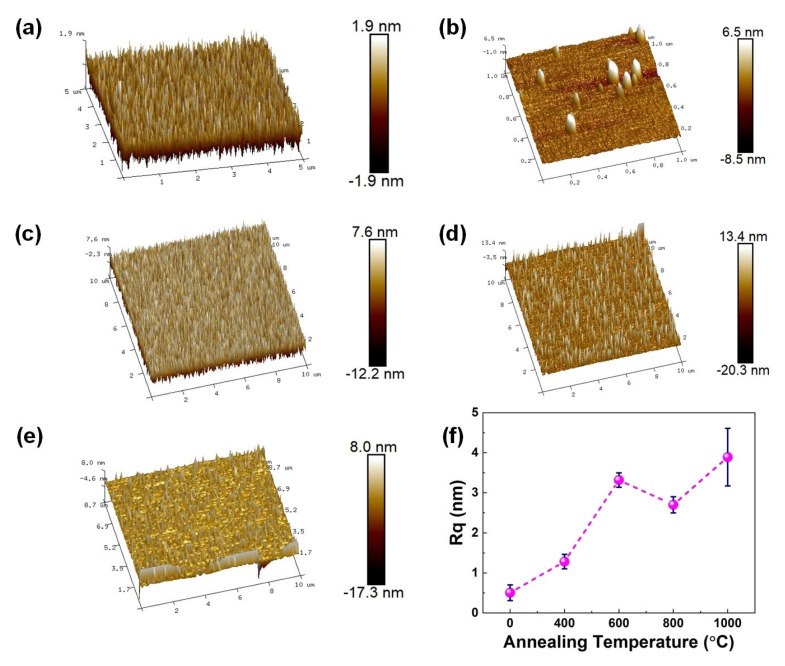
AFM surface morphologies of the GZO films with rapid annealing (**a**) as deposited, at (**b**) 400 °C, (**c**) 600 °C, (**d**) 800 °C, and (**e**) 1000 °C, and (**f**) the R_q_ changes of GZO film with rapid annealing temperature.

In order to study the effect of rapid annealing temperature on the surface morphology of Ga_2_O_3_ films, AFM was used to test the films’ roughness. The results are shown in Figure 3. The average roughness (R_q_) of GZO films annealed at 0, 400, 600, 800, and 1000 °C is 0.51, 1.28, 3.32, 2.70, and 3.89 nm, respectively. With the increase of annealing temperature, the roughness first increases, then decreases, and finally increases, which is related to the rapid annealing process and island regrowth mechanism of the film [22]. With the temperature rising from 0 °C to 800 °C, the active site of film island regrowth continues to approach saturation, the regrowth rate tends to be stable, and the roughness of the film decreases. On the contrary, at the high temperature of 1000 °C, a small number of active sites are activated, grow, and aggregate again, resulting in a faster growth rate and maximum roughness of the film.

**Figure 4:**
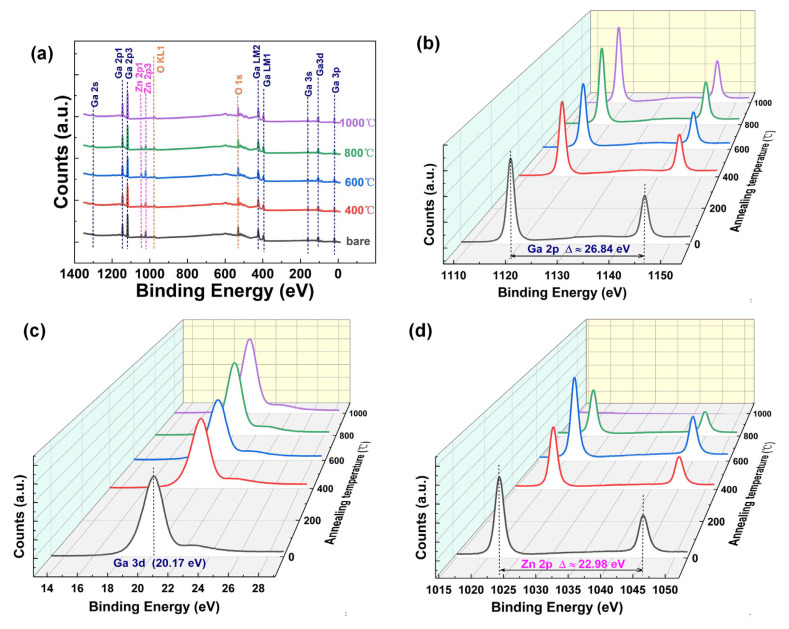
XPS spectra of GZO films after different rapid annealing temperatures. (**a**) Survey spectra, (**b**) Ga 2p spectra, (**c**) Ga 3d spectra, and (**d**) Zn 2p spectra.

Figure 4 shows the XPS spectra of GZO film at different rapid annealing temperatures. The survey spectra in Figure 4a exhibits peaks of Ga (2s, 2p, 3p, and 3d), Zn (2p), and O (1s), as well as Auger peaks from gallium (Ga LM1, LM2) and oxygen (O KL1), which is consistent with previous reports on unannealed Ga_2_O_3_ and ZnO [23,24]. In the survey spectrum, no peaks of other impurities were detected, indicating that the GZO film retained its high purity throughout the atomic layer deposition and rapid thermal annealing processes. In Figure 4b, the characteristic doublet of Ga 2p is clearly visible, with binding energies of 1144.91 eV for Ga 2p1/2 and 1118.07 eV for Ga 2p3/2, yielding an energy separation of 26.84 eV, which is consistent with previously reported values [25]. Furthermore, as depicted in Figure 4c, the peak positions of Ga 3d remained unchanged after annealing at different temperatures, suggesting that Ga atoms exhibited excellent stability after annealing. For the Zn dopant, the two peaks of Zn 2p1/2 and Zn 2p3/2 are located at 1044.97 and 1021.99 eV, respectively, with a consistent energy difference of 22.98 eV [26]. However, the peak signal of Zn2p in the sample annealed at 1000 °C is weak, implying potential transformations of ZnO at elevated temperatures, such as the formation of ZnO_2_ or carbonization [27]. Collectively, the above XPS analysis indicates that after multiple rapid high-temperature annealing cycles, the Zn element in the doped film remained relatively stable.

**Figure 5:**
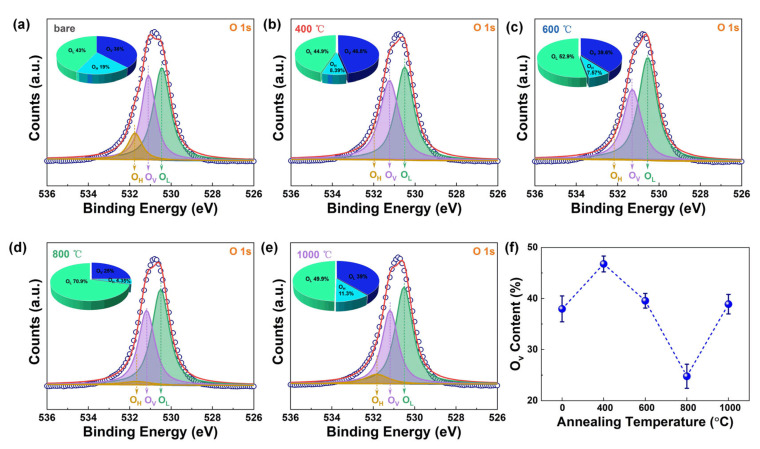
(**a**–**e**) The O 1s spectra of GZO film after different rapid annealing temperatures. The ratio of different oxygen components is obtained after calculation and shown in the inset. (**f**) The ratio of oxygen vacancy as a function of annealing temperature.

The O1s of the GZO film before and after the rapid annealing process can be divided into three peaks [24,28,29], namely, the peak of the lattice oxygen (O_L_), the peak of the oxygen vacancy (O_V_), and the peak related to the hydroxyl groups (O_H_), as shown in Figure 5a–e. The corresponding peak positions of the three types of O1s are 530.5 eV of the O_L_, 531.2 eV of the O_V_, and 531.9 eV of the O_H_. At the same time, the contents of different types of O 1s components are also calculated and shown in the corresponding insets. As the annealing temperature increases from 0 °C to 800 °C, the O_H_ content decreases from 19.0% to 4.35%, indicating that the annealing-induced growth reaction significantly purifies the film. Additionally, the surface roughness first increases and then decreases at this temperature range, further confirming the substantial improvement in film quality post-annealing. From 800 °C to 1000 °C, the O_H_ content exhibits a slight increase, which can be attributed to the crystallization process occurring at higher temperatures. Notably, even after annealing at 1000 °C, the maximum O_H_ content remains at 11.3%, substantially lower than the bare 19%. This suggests that annealing effectively reduces residual unreacted and adsorbed organic groups within the film during deposition, thereby enhancing its density, reducing surface roughness, and improving electrical properties [30].

Most notably, the oxygen vacancy (O_V_) content in GZO films exhibits a dynamic evolution during thermal annealing across different temperature regimes, as demonstrated in Figure 5f. The O_V_ concentration initially increases from 38.0% at 0 °C to a maximum of 46.8% at 400 °C, followed by a progressive decline, with further temperature elevation to 800 °C. Notably, the minimum O_V_ content of 25% is achieved at 800 °C, while subsequent annealing at 1000 °C restores the O_V_ concentration to 39%. This behavior corresponds to the characteristic growth-crystallization-regrowth transition occurring during the 0–1000 °C annealing sequence [31]. The observed O_V_ concentration fluctuations critically govern the electrical performance of the films. Particularly significant is the 34.8% reduction in O_V_ content following 800 °C annealing compared to the bare state, which correlates with enhanced electrical properties in the processed films.

**Figure 6:**
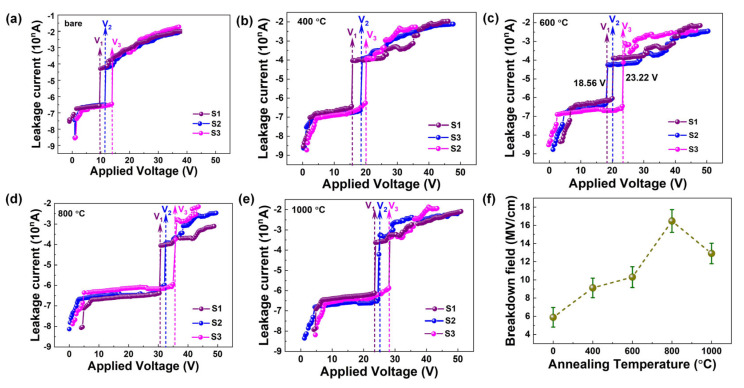
(**a**–**e**) I–V curves of the Zn-doped Ga_2_O_3_ thin films with different annealed temperatures; (**f**) the breakdown field as a function of annealed temperature.

**Figure 7:**
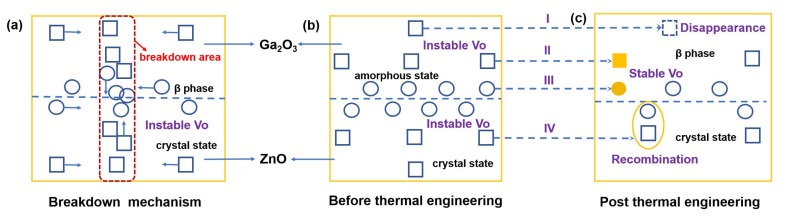
(**a**) The breakdown mechanism of GZO film, (**b**) the movement and redistribution process of oxygen vacancies before and (**c**) after thermal engineering under an external electric field.

The electrical properties of annealed Zn-doped Ga_2_O_3_ films were achieved using the MOS devices described above. The typical I–V characteristic curves of the annealed Zn-doped Ga_2_O_3_ film-based MOS structure are shown in Figure 6a. At 0.5 V, the leakage current of all devices was less than 5.5 × 10^−9^ A, which suggests micro Zn-doped Ga_2_O_3_ maintains good insulation properties. When the applied voltage gradually increases to the breakdown voltage, the leakage current of the device suddenly increases, which accords with the hard breakdown characteristic of the device. The Zn-doped Ga_2_O_3_ film prepared by ALD has the characteristics of high density, and the regrowth of crystal during annealing will affect the electrical properties of the device. This is different from the soft breakdown characteristics of bare Ga_2_O_3_-based films [32]. The average breakdown voltage fields are 5.88, 9.12, 10.31, 16.47, and 12.90 MV/cm for Zn-doped Ga_2_O_3_ film with annealed temperatures of 0, 400, 600, 800, and 1000 °C, respectively. The average breakdown field of MOS devices based on Zn-doped Ga_2_O_3_ films with different annealing temperatures are listed in Table S2. By increasing the annealed temperature, the *E_g_* value decreases, which is the main intrinsic breakdown voltage [33]. On the other hand, the main breakdown characteristics are due to the internal structural characteristics of the film. Accordingly, by taking into account the film thickness, the breakdown electric field of the film was calculated and is shown in Figure 6b. Similar to the variation of breakdown voltage, the breakdown field strength first increases and then decreases with the increase of annealing temperature. This could be due to the changes of oxygen vacancies and defects in internal Ga_2_O_3_ and ZnO. Under a certain electric field strength, internal defects and vacancies can move and rearrange, and then act as conductive channels, making the film more easily broken down, as exhibited in Figure 7a. The concentration of oxygen defects, as shown in Figure 5, first increases, then decreases, and finally increases with the annealing temperature increasing. The GZO film annealed at 800 °C has the lowest concentration of oxygen defects and thus requires a larger breakdown electric field. Additionally, the breakdown field strength of the GZO film annealed at 800 °C is higher than that of Ga_2_O_3_ and ZnO [34,35]. This indicates that trace Zn doping and annealing engineering can significantly enhance the electrical properties of Ga_2_O_3_, promoting its potential application in wide bandgap and high-power devices.

Figure 7a shows the breakdown mechanism of GZO thin film. As described above, under the action of an electric field, oxygen vacancies and other defect states aggregate with each other to form conductive channels, thus causing the thin film to be broken down under a certain voltage [36]. After annealing, obvious changes occurred inside the GZO film that increased the breakdown voltage, as shown in Figure 7b,c. First, the unstable defects in Ga_2_O_3_ and ZnO grow again after annealing, thus reducing the density of the defect states. Second and third, after high-temperature annealing, the unstable oxygen vacancy in the interior and at the interface will be transformed into a stable oxygen vacancy so that the carrier concentration involved in the breakdown will be reduced. Fourth, the defect states with different charge characteristics will also compound with each other, forming faults and failing to form a complete conductive channel. In addition, oxygen vacancies create mid-gap states that facilitate trap-assisted tunneling or impact ionization, lowering the effective breakdown field by providing pathways for premature current flow. Further, the interfaces between different phases can concentrate electric fields, accelerating carrier multiplication. All of the above significantly reduced internal defect states of GZO films caused by annealing and significantly enhanced the breakdown characteristics of GZO films.

## 4. Conclusions

In this study, Zn-doped Ga_2_O_3_ thin films with optimized post-deposition thermal engineering were successfully fabricated through ALD followed by rapid thermal annealing. The amorphous GZO films with desirable morphological and structural characteristics were achieved through precise control of the layer-by-layer ALD deposition process. Subsequent thermal treatment within the 400–1000 °C range induced controlled crystallization of the GZO films. Notably, the 800 °C annealed GZO demonstrated an enhancement in the breakdown field compared to both as-deposited films and those annealed at other temperatures. The electrical property of films is closely related to the behavior of the internal oxygen vacancy. The lower oxygen vacancy concentration and excellent structure characteristics of GZO annealing at 800 °C significantly improved the breakdown characteristics of the MOS device. Moreover, the breakdown mechanism of GZO thin film was also discussed in detail. The developed GZO material system demonstrates significant potential for advanced optoelectronic applications, particularly in UV-C/UV-B flexible photodetectors, as well as in high-power electronic devices through implementation in n-n junctions and heterojunction transistors.

## Data Availability

Data are contained within the article and Supplementary Materials.

## Supplementary Materials

The following supporting information can be downloaded at: https://www.mdpi.com/article/10.3390/nano15070499/s1, Figure S1: XRD patterns of the grown Ga_2_O_3_, ZnO, and GZO films grown on Si (100) substrate; Table S1: Summary of the details of the parameters for one growth supercycle during the reaction process, estimated and measured thickness of GZO films; Table S2: The breakdown voltages and average breakdown field of MOS devices based on Zn-doped Ga_2_O_3_ films with different annealing temperature. The experimental methods of ALD deposition of GZO and MOS device fabrication and measurement are also described in the Supplementary Materials.
