# Supplementary material for: Effect of Rapid Thermal Annealing on the Characteristics of Micro Zn-Doped Ga2O3 Films by Using Mixed Atomic Layer Deposition
Source: Nanomaterials (Basel). 2025 Mar 26;15(7):499. doi: 10.3390/nano15070499 (PMC11990894; doi:10.3390/nano15070499)
Supplement: Supplementary file 1 [file nanomaterials-15-00499-s001.zip › nanomaterials-3517469-supplementary.pdf]

Supporting Information

Effect of Rapid Thermal Annealing on the Characteristics of micro Zn-Doped Ga_2O_3 Films by Using Mixed Atomic Layer Deposition

Jiajia Tao[1, 2, 3], Xishun Jiang[4, *], Aijie Fan[2], Xianyu Hu[3], Ping Wang[4], Zuoru Dong[1], Yingjie Wu[1]

[1]*No. 50 Research Institute of China Electronics Technology Group Corporation, Shanghai 200331, China*

[2]*Zhangjiang Laboratory, Shanghai 201210, China*

[3]*State Key Laboratory of ASIC and System, School of Microelectronics, Fudan University, Shanghai 200433, China*

[4]*School of Mechanical and Electronical Engineering, Chuzhou University, Chuzhou 239000, China*

Email: jxs@chzu.edu.cn (X.S. Jiang, corresponding author).

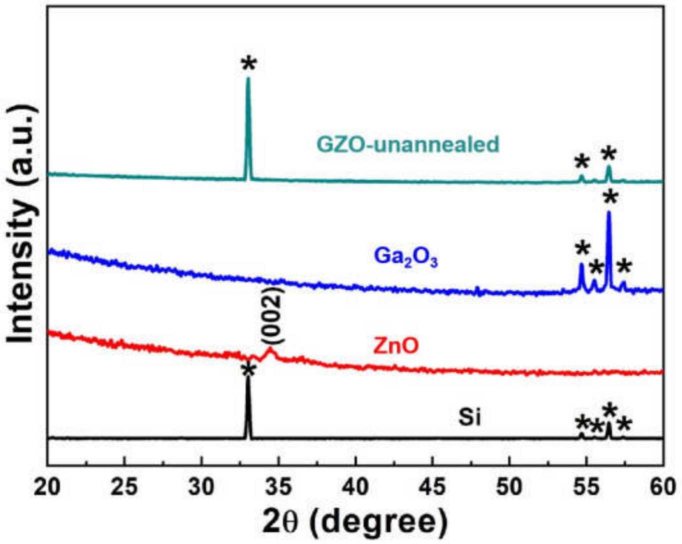

Figure S1 XRD patterns of the grown Ga_2O_3, ZnO, and GZO films grown on Si (100) substrate

Table S1 Summary of the details of the parameters for one growth supercycle during the reaction process, estimated and measured thickness of GZO films

Sample	*Number of Ga_2O_3*	*Number of ZnO*	*Number of supercycle*	*Estimated thickness (nm)*	*Measured thickness (SE, nm)*	*Estimated GPC (Å/cycle)*	*Experimental GPC (Å/cycle)*
**Ga_2O_3**	300	0	300Ga_2O_3	/	18	/	0.6
**ZnO**	0	100	100ZnO	/	20	/	2
**Ga/Zn=7:1**	210	30	33(7Ga_2O_3+1ZnO)	20.46	25.08	6.2	7.6

Table S2 The breakdown voltages and average breakdown field of MOS devices based on Zn-doped Ga_2O_3 films with different annealing temperature

Annealing temperature **(°C)**	***Breakdown voltages*（V）**			***Average breakdown field*** **(MV/cm)**
	S1	S2	S3	
0	11.66	9.68	13.96	5.88
400	15.82	18.92	19.96	9.12
600	18.56	20.20	23.09	10.31
800	32.54	30.68	35.61	16.47
1000	25.27	23.85	28.28	12.90

Experimental Methods

1) ALD Deposition of GZO

The TH-ALD and PE-ALD were employed for the growth of ZnO and Ga_2O_3 films, respectively. The susceptor temperature T_s was fixed at 200 °C for the deposition of all films unless stated otherwise. The precursors of TMGa was maintained at 13 °C whereas the DEZn and water were kept at room temperature (20 °C). Ar was employed as both the carrier and purge gas during the experiment. The flow rate of Ar was modulated by a mass flow controller (MFC). For the remote plasma process, the O_2 flow was set at 50 sccm, a pressure of 15 mTorr, and a RF power of 200 W. The PE-ALD Ga_2O_3 growth sequence is composed of 18 ms of TMGa exposure, 2 s of Ar purging, 4.5 s of O_2 plasma exposure, and 2 s of Ar purging. Moreover, the typical TH-ALD sequence of ZnO consists of several sequential steps including 0.25 s of DEZn exposure, 3 s of Ar purging, 0.2 s of reactant exposure, and 2 s of Ar purging. For one supercycle of Zn-doped Ga_2O_3 (GZO) deposition, 1 cycle of ZnO was performed after 7 cycles of Ga_2O_3.

2) MOS Device Fabrication & Measurement

Zn-doped Ga_2O_3 films with varying annealing temperatures were sequentially deposited on Si substrates at 200 °C using a BENEQ TFS200 ALD system. Metal oxide semiconductor (MOS) capacitors were constructed for in-depth electrical characterization. Magnetron sputtering was used to deposit Cr/Au electrodes with a thickness of 200 nm on the annealed Zn-doped Ga_2O_3 films and the bottom of the Si substrate. The top electrodes were 200 μm wide square shaped arrays. A Keithley 4200 parameter analyzer was used to perform leakage current-voltage (I-V) tests on the Zn-doped Ga_2O_3 devices in the air at room temperature on the Lakeshore probe station. The voltage range was 0-50 V with a step of 0.1 V.
